# Panniculectomy Combined with Bariatric Surgery by Laparotomy: An Analysis of 325 Cases

**DOI:** 10.1155/2015/193670

**Published:** 2015-11-15

**Authors:** Vincenzo Colabianchi, Giancarlo de Bernardinis, Matteo Giovannini, Marika Langella

**Affiliations:** ^1^Plastic Surgery Unit, Casa di Cura Villa Alba, Bologna Medical Center, 40136 Bologna, Italy; ^2^General Surgery Unit, Presidio Ospedaliero Villa Letizia, 67100 L'Aquila, Italy; ^3^General Surgery Unit, Casa di Cura Villa Alba, Bologna Medical Center, 40136 Bologna, Italy

## Abstract

Surgical treatment of obese patients is much debated in the literature because of the significant intraoperative risks related to comorbidities presented by this type of patients. Recent literature suggests that panniculectomy should follow bariatric surgery after the patient's weight loss has been stabilized. However, when performed by laparotomy, bariatric surgery can be combined with panniculectomy. This paper presents the analysis of 325 cases of patients undergoing abdominal panniculectomy combined with bariatric surgery. The study highlights the risks, complications, and benefits of the combined procedure and describes a standardized technique for excision of a large abdominal panniculus in a short operating time.

## 1. Introduction

Obesity and overweight are recognized as one of the most important public health problems. In recent years, bariatric surgery has become increasingly popular and a valid alternative to dieting for patients with morbid obesity [1, 2]. Patients who are candidates for obesity surgery often exhibit a moderate to large panniculus (grades 1 to 5), according to the classification by Igwe Jr. et al. [3, 4]. A large pendulous abdomen can affect mobility, limits physical activity, affects personal hygiene, and has a negative impact on the professional life of patients [5]. A sagging abdomen, which is exacerbated after marked postbariatric weight loss, also may cause lymphedema of the abdominal wall, which may limit the social and sexual life of the patient and lead to negative psychological consequences and depression [6]. Moreover, after bariatric surgery by laparotomy, patients are left with an unsightly permanent scar from the xiphoid process to the umbilicus, which may be distressing, especially for younger women.

Recent literature suggests that panniculectomy should be performed separately from bariatric surgery and only after the weight loss of the patient has been stabilized [2, 7, 8]. However, to prevent these conditions during the weight-loss period, bariatric surgery can be combined with abdominal panniculectomy if prolonged operating time and excessive blood loss are avoided [4, 5, 9].

The authors report a detailed description of the results of 325 patients treated by combining bariatric surgery with immediate panniculectomy.

## 2. Patients and Methods

Between January 2008 and February 2014, 325 patients (171 women and 154 men), with morbid obesity, underwent bariatric surgery combined with abdominal panniculectomy. The patients were operated on at the Plastic Surgery Unit of the Casa di Cura Villa Alba (Bologna, Italy) and at the General Surgery Unit of the San Pierdamiano Hospital (Faenza Ravenna, Italy), by the same surgical team. The study was performed in accordance with the ethical standards of the 1964 Declaration of Helsinki and its subsequent amendments. Written informed consent was obtained from all patients prior to their inclusion in the study.

The patients had a mean age of 41.5 ± 9.1 years (range: 18 to 65 years) and mean body mass index (BMI) of 59.3 ± 10.4 kg/m (range: 52.7 to 69.4 kg/m), with a mean overweight of 243%. BMI distribution among the study cohort is shown (Table 1).

The severity of the pendulous abdomen was classified into five grades, according to Van Hout et al. [6]. Most patients (69%, *n* = 225) had panniculus grades 2 and 3 (Table 2). Eighteen patients (5.5%) had pendulous abdomen complicated by lymphedema. The following comorbidities, obtained directly from the medical record and the patient's history, were recorded: hypertension (65%); diabetes (45%), obstructive sleep apnea syndrome (OSAS) (36%); hypercholesterolemia (31%); chronic obstructive pulmonary disease (COPD) (25%), depression (24%); asthmatic bronchitis (18%); arthralgia (16%); gastroesophageal reflux disease (GERD) (15%); and chronic ischemic heart disease (12%) (Table 3).

All patients included in this study were initially evaluated by a multidisciplinary team before undergoing bariatric surgery by laparotomy combined with abdominal panniculectomy.

Patients with central obesity, BMI greater than 70 kg/m, severe cardiorespiratory disease, and incisional hernias in subcostal or xiphopubic scars, and those in whom an excessive stress on the final suture line was predicted with the pinch test were excluded from the study.

The complications arising from the combination of bariatric surgery with panniculectomy were grouped into two three-year periods. This distinction in two periods has been made to take into account the experience of the surgical team.

### 2.1. Preoperative Marking and Management

The amount of tissue to be resected was assessed by the pinch test with the patient standing up and then in a semiflexed position (30°) on a bed. A vertical line extending from the xiphoid process to the symphysis pubis and passing over the umbilicus was marked. The lateral limits of the diamond-shaped area of skin and adipose tissue to be resected were marked with the patient in a sitting position. Horizontal lines were marked, using the abdominal fold as the lower limit and the upper edge of the umbilicus as the upper limit of the lateral marks. If the skin was irritated or infected, the inferior line was moved distally to the healthy skin. Finally, two vertical paramedian lines were marked bilaterally to the xiphopubic line to ensure symmetrical resection of the adipose tissue, and a line defining the position of the costal arches was traced. Before surgery, all patients received antithromboembolic prophylaxis, including the wearing of compression stockings and administration of dalteparin sodium, combined with antibiotic prophylaxis with third-generation cephalosporins.

### 2.2. Surgical Procedures

The operation was performed by the same surgical team and with the aid of mechanical means for lifting adipose tissue.

**Figure 1:**
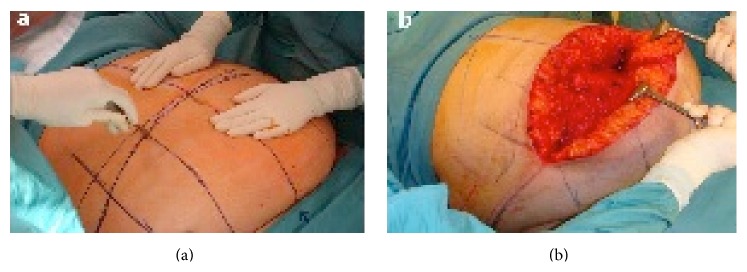
(a) Incision made in the medial two-thirds of the upper horizontal line. (b) Operative field. Mobilization of the dermal-fat flap from top to bottom.

An incision was made in the medial two-thirds of the upper horizontal line with a cold blade scalpel (Figure 1(a)). The subcutaneous tissue was dissected with an electrocautery in spray mode at 35% intensity to limit heat damage to the adipose tissue. The incision was deepened until Scarpa's fascia was exposed and extended laterally to the muscle fascia on the midline. Special attention was given to the umbilical stump, which was usually buried. This maneuver allows direct access to the periumbilical perforators, which have a considerably large caliber in obese patients, permitting meticulous hemostasis and reduction of blood loss. The dermal-fat flap was elevated with limited undermining of the supraumbilical and epigastric regions and extended superiorly to the xiphoid process. The lateral undermining of the abdominal flap toward the costal arch should be avoided as much as possible to minimize dead space and prevent damage to the abdominal vasculature. The described dissection, although limited, allows the creation of an operative field sufficient to perform the entire procedure.

Proceeding cautiously, the panniculus adiposus was undermined from the muscle fascia up to the suprapubic line. In order not to skeletonize the muscle fascia, a layer of adipose tissue was left, so that part of the lymphatic system was preserved, thus reducing the risk of seroma formation. This maneuver can be facilitated by placing the patient in the anti-Trendelenburg position, rotating the operating table by 180°, and mobilizing the flap from top to bottom (Figure 1(b)). Then, the patient was placed in the semirecumbent position (30° between the trunk and legs), allowing the evaluation and better control of the exact amount of tissue to be diathermied to ensure that the incision is closed without excessive tension on the suture line. The skin incisions were completed bilaterally on the upper horizontal line and along the entire length of the lower horizontal line. Next, the subcutaneous tissue was dissected down to near the fascia, starting from the sides of flap and proceeding medially. In the suprapubic region, a 2 to 3 cm thick layer of adipose tissue was not removed to prevent scar contracture or formation of atrophic scars. The abdominal panniculus was removed and weighed (to make the necessary adjustments to correct for postoperative infusion therapy).

The median laparotomy between the xiphoid process and the umbilicus allows biliary-intestinal bypass to be performed. Closure of abdominal wall was carried out in two layers: one for the parietal peritoneum and one for the fascia. Hemostasis was accurately controlled, the abdominal wall was washed with antibiotic solution, and one suction drain (caliber 21) was placed on each side.

Umbilical reconstruction involved a double-Y incision of the abdominal skin. The umbilicus was sutured with Gillies stitches using 4-0 nylon suture (avoiding its fixation to the muscle fascia), leaving a stump of length similar to the thickness of the dermal-fat flap. This preventive measure is useful to reduce the mechanical stress on the umbilicus, which is already burdened by the partial devascularization caused by the undermining of the skin.

Eight to ten stitches were placed fixing the Scarpa's fascia to the muscle aponeurosis to close the dead space. The subcutaneous tissue was closed in three layers with absorbable 2-0 and 3-0 vicryl suture. Closure of the skin was then performed with intradermal sutures for patients with panniculus grade 1 or 2 and metal staples for patients with panniculus grade 3, 4, or 5. A compressive dressing was then applied and maintained in place for 60 days.

The mean operating time for the different steps of abdominal panniculectomy combined with bariatric surgery was 45 minutes for tissue dissection, 75 minutes for bariatric procedures, and 1 hour for abdominal closure, for a total of 3 hours.

## 3. Results

The mean redundant panniculus excised was 6.5 kg (range: 3.2–16.5 kg).

Patients who had panniculus grade 1 or 2 had the drains removed before hospital discharge on postoperative day 7, and for those who had panniculus grade 3, 4, or 5, the drains were removed on postoperative day 14.

**Figure 2:**
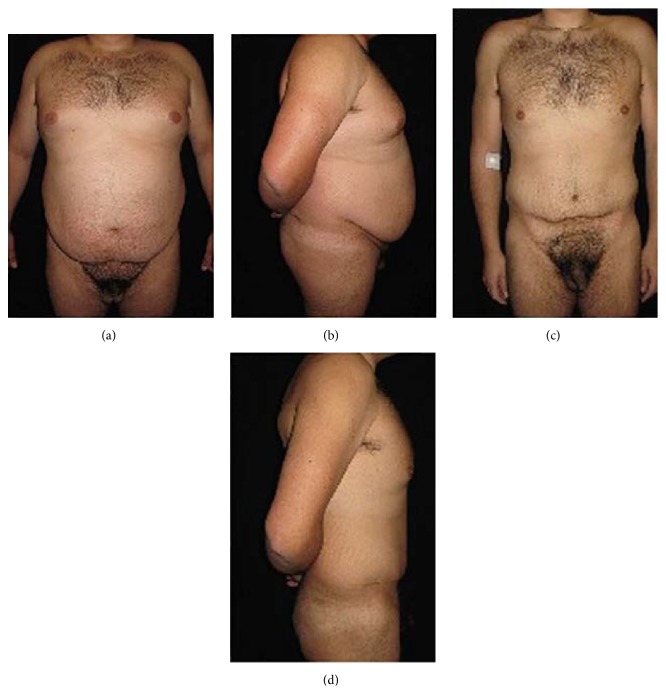
(a) Preoperative frontal view and (b) lateral view of a male patient (BMI = 44 kg/m^2^; body weight = 136 kg). (c) Postoperative frontal view and (d) lateral view five years after surgery (body weight = 73 kg).

**Figure 3 fig3:**
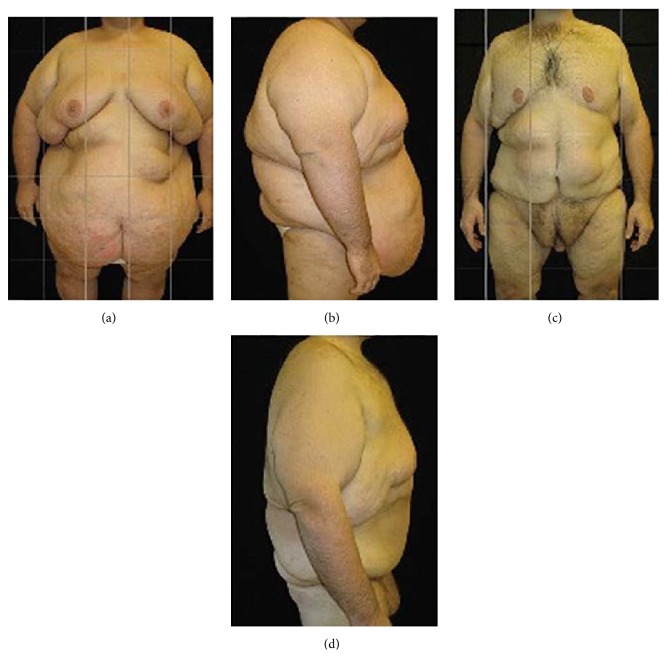
(a) Preoperative frontal view and (b) lateral view of a male patient (BMI = 69.9 kg/m^2^; body weight = 220 kg). (c) Postoperative frontal view and (d) lateral view five years after surgery (body weight = 110 kg).

The mean hospital stay was 8 days (range: 5–13 days). Complications associated with panniculectomy (Table 4) and those associated with bariatric surgery (Table 5) were grouped into two three-year periods. A marked decrease in the incidence of complications occurred during the second period of 3 years as shown in Figures 2 and 3. This result shows how the practical experience of the team in the management of this type of patients is essential.

It was observed that 23.5% of patients had hernias or incisional hernias; 78% of these hernias were repaired with direct suture and 22% with surgical mesh. Two patients (0.61%) died of acute myocardial infarction and one (0.31%) due to septicemia, caused by anastomotic dehiscence.

Apart from a few exceptional cases, patients who underwent panniculectomy did not require further aesthetic and nonsurgical procedures. Seromas were treated with antibiotics and fluid collection drainage on an outpatient basis.

**Figure 4 fig4:**
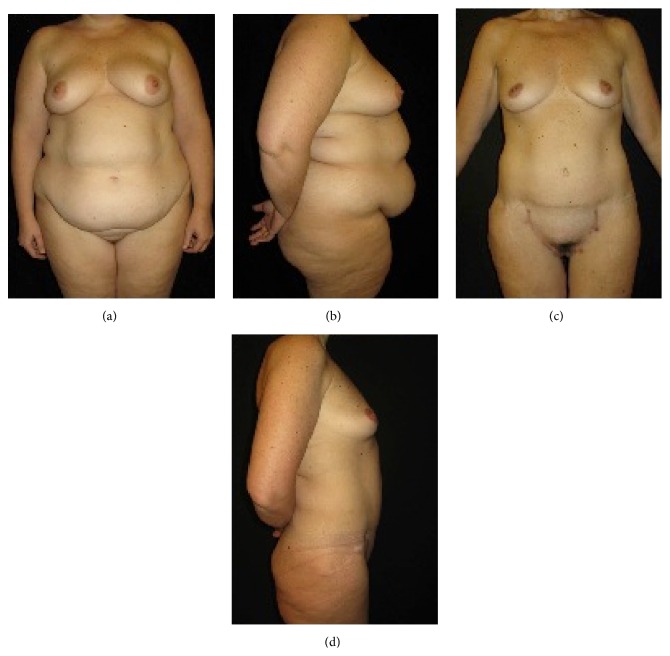
(a) Preoperative frontal view and (b) lateral view of a female patient (BMI = 50 kg/m^2^; body weight = 135 kg). (c) Postoperative frontal view and (d) lateral view three years after surgery (body weight = 65 kg).

**Figure 5 fig5:**
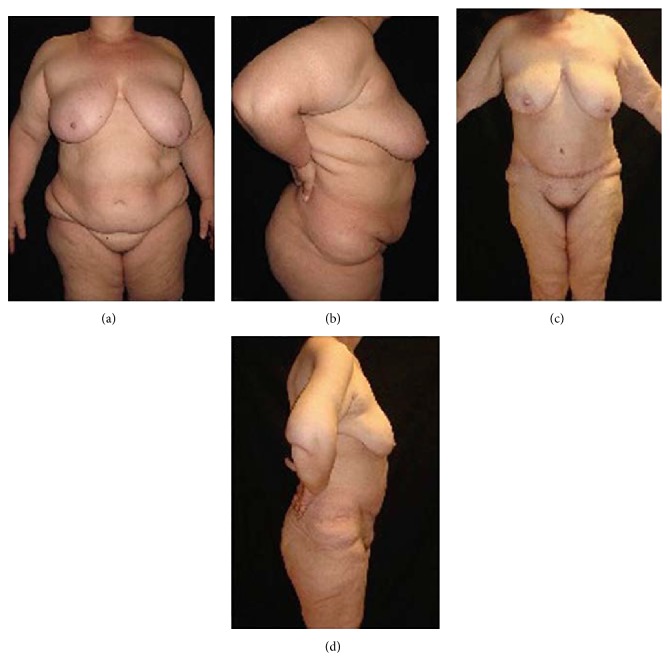
(a) Preoperative frontal view and (b) lateral view of a female patient (BMI = 39 kg/m^2^; body weight = 106 kg). (c) Postoperative frontal view and (d) lateral view 11 months after surgery (body weight = 71 kg).

**Figure 6 fig6:**
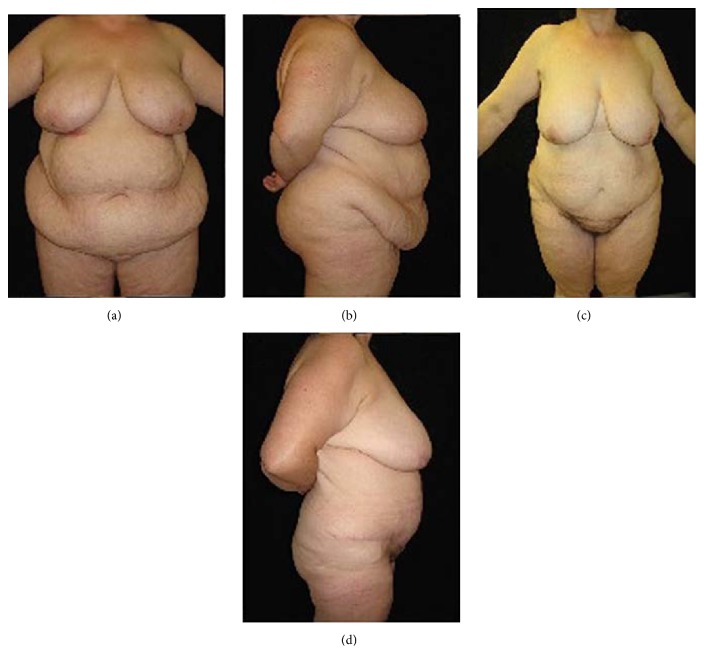
(a) Preoperative frontal view and (b) lateral view of a female patient (BMI = 46 kg/m^2^; body weight = 129 kg). (c) Postoperative frontal view and (d) lateral view five years after surgery (body weight = 82 kg).

**Figure 7 fig7:**
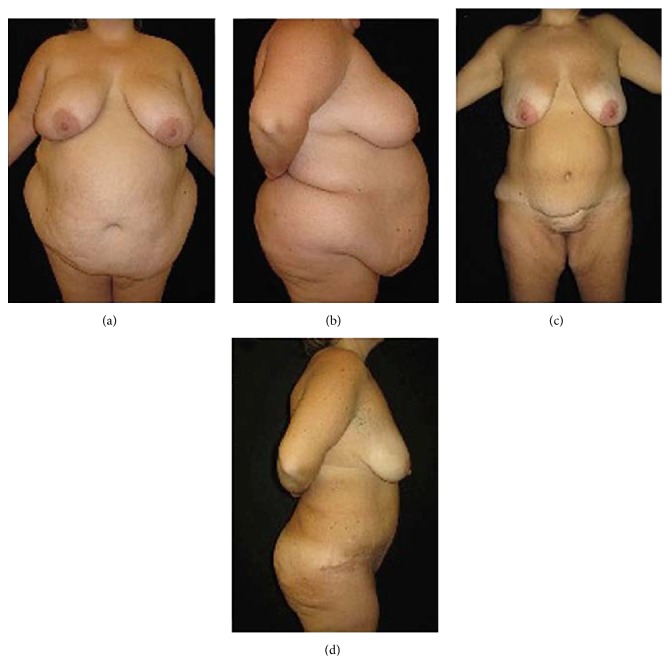
(a) Preoperative frontal view and (b) lateral view of a female patient (BMI = 55 kg/m^2^; body weight = 147 kg). (c) Postoperative frontal view and (d) lateral view five years after surgery (body weight = 67 kg).

**Figure 8:**
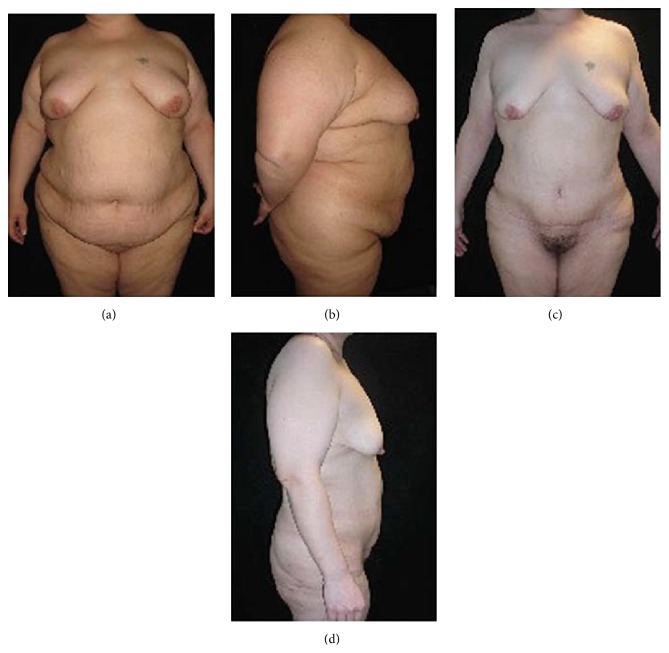
(a) Preoperative frontal view and (b) lateral view of a female patient (BMI = 45 kg/m^2^; body weight = 133 kg). (c) Postoperative frontal view and (d) lateral view 18 months after surgery (body weight = 72 kg).

Typical examples of patients treated with bariatric surgery combined with panniculectomy are shown (Figures 2 [Fig fig3] [Fig fig4] [Fig fig5] [Fig fig6] [Fig fig7]–8).

## 4. Discussion

This study, conducted on 325 patients, applying a combined approach of bariatric surgery and panniculectomy, shows that benefits, including immediate reduction of the abdominal apron and prevention of the worsening of this condition during weight loss; no visible scarring; improved personal hygiene and resolution of dermatological problems; prevention of lymphedema of the abdominal wall during the weight loss period, as the exacerbation of the abdominal ptosis may lead to lymphatic and venous stasis; improved quality of life and self-esteem and weight loss, can be achieved. Also, one of our main concerns was to develop a surgical strategy for elimination of postlaparotomy scars, especially in younger women.

The correction of the resection plane of the diamond-shaped panniculus, meticulous hemostasis, adequate compression during the postoperative period, and use of subcutaneous drains during an appropriate period of time resulted in a marked reduction of specific immediate complications, including bleeding, seroma formation, infection, and wound dehiscence. The postoperative complications of this large sample of 325 patients were evaluated in two successive 3-year periods to assess the practical effects of increasing surgical experience over time. At the first 3 years, the present study was characterized by large incidence rates of postoperative complications, which gradually decreased (Figures 2 and 3). The incidence rates of postoperative complications in the second 3-year period of the study were similar to those reported by Igwe Jr. et al. [4] for both combined and postbariatric panniculectomy (Table 6). Therefore, the surgical experience acquired during the study period decreased operating time and hospital stay, motivating us to extend the indications of panniculectomy combined with bariatric surgery by laparotomy.

At present, the literature suggests that abdominal lipectomy should be performed at a later stage to bariatric surgery, after weight loss has been stabilized. It has been reported that abdominoplasty should be conducted up to two years after the bariatric surgery [2, 7, 8]. In our country, as in other countries, the time between the two operations can be much longer because of economic reasons, and this long wait may lead to depression, stress, and poor quality of life of the patient [6].

Some patients who did not undergo panniculectomy combined with bariatric surgery (because they were excluded from the study due to severe comorbidities) had abdominal lymphedema during the weight loss period after bariatric surgery with involvement of the lower limbs. There was a particular case in which absorption of a lymphedema (which reached extraordinary dimensions and weighed about 70 kg) after bariatric surgery required a long outpatient treatment with diuretics before the panniculus could be successfully removed.

The bariatric technique used in this study falls into the category of malabsorptive procedures. This technique led to a stable weight reduction without dietary restrictions in this large case series. Advances in laparoscopic techniques and instrumentation have enabled surgeons to perform bariatric operations for morbid obesity laparoscopically [2]. The laparoscopic approach reduces postoperative pain, decreases complications, shortens length of hospital stay, and allows an early return of the patient to work [10]. Laparoscopy provides benefits, such as reduced operative trauma and decreased risk of incisional hernia and postoperative wound infection [11]. Bariatric surgery combined with panniculectomy may be performed using a laparoscopic approach. Further studies combining panniculectomy with laparoscopic bariatric surgery are necessary for comparison of results obtained by the different techniques.

Over the years we have performed this procedure in patients with pendulous abdomen of different grades [4]. Based on the feedback from surgeons, we hypothesized that there are other anthropometric and physiological parameters that are also important to be evaluated during surgery and postoperative period, including the frontal projection of the abdominal apron on the thoracoabdominal wall; the lateral projections of the abdominal apron on the anterior axillary lines (2 measuring points); plicometry under the midline and midclavicular line (3 measuring points); and the degree of absorption of the abdominal panniculus. The use of these seven parameters, which complement the classification by Igwe Jr. et al. [4], allows the creation of a scoring system that can anticipate the difficulty of the surgical procedures and risks of early postoperative complications.

The patients who participated in this study, except in few exceptional cases, did not require aesthetic and nonrevision surgeries because of the subsequent weight loss. In addition, the patient is hospitalized and undergoes general anesthesia only once.

The development of the described technique combining panniculectomy with bariatric surgery allowed us to significantly extend the indications of this treatment, with very good results both in the short and long terms.

## 5. Conclusions

The retrospective study of 325 patients with morbid obesity who underwent panniculectomy combined with bariatric surgery between 2008 and 2014 indicated that removal of adipose tissue improves the patient's quality of life during the weight-loss period and prevents all the disabling complications, including lymphedema. These complications would make postbariatric panniculectomy necessary, with increased risks to the patient. Incidence rates of postoperative complications associated with the combined surgical treatment were similar to those of postbariatric panniculectomy. With adequate preparation, the combined treatment can be extended to patients with morbid obesity and pendulous abdomen complicated by lymphatic stasis. The patients who participated in this study, except in few exceptional cases, did not require revision surgeries. Accordingly, we believe that the combined technique described in this paper is not only a suitable alternative treatment for economic reasons, but also a sound choice that improves postoperative aesthetic and functional outcome.

## Figures and Tables

**Table 1:** Body mass index (BMI) distribution among the study cohort.

BMI (Kg/m^2^)	Woman (*n*)	Men (*n*)	Total (*n*)
38.9–50	7	5	12
50.1–55	10	9	19
55.1–60	84	59	143
60.1–65	60	56	116
65.1–70	10	25	35
Total	**171**	**154**	**325**

**Table 2:** Distribution of panniculus grading [9] in the study cohort (*n* = 325).

Grades	Description	*N*
Grade 1	Panniculus covers pubic hairline but not the entire mons pubis	31
Grade 2	Panniculus extends to cover the entire mons pubis	101
Grade 3	Panniculus extends to cover the upper thigh	124
Grade 4	Panniculus extends to mid-thigh	61
Grade 5	Panniculus extends to the knee and beyond	8

**Table 3:** Comorbidities of patients (*n* = 325).

Comorbidities	
OSAS* *	36%
Depression	24%
GERD* *	15%
NIDDM* *	38% (84% severe diabetes)
IDDM* *	7% (16% severe diabetes)
COPD	25%
Arthralgia* *	16%
CV pathologies	12%
Hypertension	65%
Hypercholesterolemia	31%
Asthmatic bronchitis	18%

OSAS: obstructive sleep apnea syndrome; GERD: gastroesophageal reflux disease; NIDDM: non-insulin-dependent diabetes mellitus; IDDM: insulin-dependent diabetes mellitus; COPD: chronic obstructive pulmonary disease; CV pathologies: cardiovascular pathologies.

**Table 4:** Postoperative complications for the 325 patients during the study period associated with panniculectomy.

Postoperative complications, *n* (%)	2007–2013 (*N* = 325)	2007–2010 (*N* = 164)	2010–2013 (*N* = 161)
Seroma	59 (18.2)	51 (31.1)	8 (5.0)
Dehiscence	44 (13.5)	31 (18.9)	13 (8.1)
Dysesthesia/anesthesia	33 (10.2)	17 (10.4)	16 (9.9)
Liponecrosis	29 (8.9)	19 (11.6)	10 (6.2)
Superficial edema* *	19 (5.8)	13 (7.9)	6 (3.7)
Hemorrhage	17 (5.2)	12 (7.3)	5 (3.1)
Cardiorespiratory complications	16 (4.9)	11 (6.7)	5 (3.1)
Skin necrosis	13 (4.0)	10 (6.1)	3 (1.9)
Loss of the umbilicus	1 (0.3)	1 (0.6)	0 (0)

**Table 5:** Postoperative complications for the 325 patients during the study period associated with the bariatric surgery.

Postoperative complications, *n* (%)	2007–2013 (*N* = 325)	2007–2010 (*N* = 164)	2010–2013 (*N* = 161)
Incisional hernia	59 (18.2)	38 (23.2)	
Respiratory insufficiency	44 (13.5)	31 (18.90)	13 (8.07)
DVT/PE* *	33 (10.2)	25 (15,24)	8 (4.9)
Surgical infection	29 (8.9)	24 (14,63)	5 (3.1)
Intestinal occlusion	19 (5.8)	16 (9.7)	3 (1.8)
Hemoperitoneum	17 (5.2)	11 (6.7)	6 (3.7)
Anastomotic dehiscence	16 (4.9)	12 (7.3)	4 (2.4)
Renal failure	13 (4.0)	9 (5.4)	4 (2.4)
Intraoperative death	1 (0.3)	1 (0.6)	0 (0)

DVT/PE: deep vein thrombosis and pulmonary embolism.

**Table 6:** Comparison of postoperative complications found in this study in the first and second 3-year periods with those reported in a previous study [9].

Postoperative complications	First 3 years (*N* = 164)	Second 3 years (*N* = 161)	Combined treatment (*N* = 428)	Postbariatric panniculectomy (*N* = 149)
Dehiscence* *	18.9%	8.1%	9.8%	7.4%
Seroma	31.1%	5.0%	4.2%	4.7%
Hemorrhage	7.3%	3.1%	1.9%	1.3%
DVT/PE	4.9%	0.6%	—	—
Incisional hernia	23.2%	6.8%	—	—
Skin necrosis	6.1%	1.9%	—	—
Death	0.6%	0%	—	—
